# Supplementary material for: Arabidopsis G-Protein β Subunit AGB1 Negatively Regulates DNA Binding of MYB62, a Suppressor in the Gibberellin Pathway
Source: Int J Mol Sci. 2021 Jul 31;22(15):8270. doi: 10.3390/ijms22158270 (PMC8347620; doi:10.3390/ijms22158270)
Supplement: Supplementary file 1 [file ijms-22-08270-s001.zip › ijms-1253262-supplementary.pdf]

Supporting Information

Fig. S1 Verification of mutants and transgenic plants.

Fig. S2 Phenotypes of wild type (Col-0) and *agb1-2* under 10 μM GA_3.

Fig. S3 Phenotypes of wild type, *agb1-2*, and *N692967* under 1 μM GA_3 treatment at 1d, 7d, and 10d.

Fig. S4 Phenotypes of wild type, *agb1-2*, and *N692967* under 10 μM GA_3 treatment at 1d, 7d, and 10d.

Fig. S5 Phenotypes of wild type, *agb1-2*, and *N692967* under 100 μM GA_3 treatment at 1d, 7d, and 10d.

Fig. S6 Phenotypes of wild type, *agb-1-2*, *N692967*, *MYB62:GFP/WT-8*, *MYB62:GFP/WT-10*, *MYB62:GFP/agb1-2-1*, and *MYB62:GFP/agb1-2-4* under 1 μM GA_3 treatment at 1d, 7d, and 10d.

Fig. S7 Phenotypes of wild type, *agb-1-2*, *N692967*, *MYB62:GFP*/WT-8, *MYB62:GFP/WT-10*, *MYB62:GFP/agb1-2-1*, and *MYB62:GFP/agb1-2-4* under 10 μM GA_3 treatment at 1d, 7d, and 10d.

Fig. S8 Phenotypes of wild type, *agb-1-2*, *N692967*, *MYB62:GFP/WT-8*, *MYB62:GFP/WT-10*, *MYB62:GFP/agb1-2-1*, and *MYB62:GFP/agb1-2-4* under 100 μM GA_3 treatment at 1d, 7d, and 10d.

Fig. S9 Phenotypic identification of wild type, *agb-1-2*, *N692967*, *MYB62:GFP/WT-8*, *MYB62:GFP/WT-10*, *MYB62:GFP/agb1-2-1* and *MYB62:GFP/agb1-2-4* under 1 μM GA_3 treatment at 10d.

Fig. S10 Phenotypic identification of wild type, *agb-1-2*, *N692967*, *MYB62:GFP/WT-8*, *MYB62:GFP/WT-10*, *MYB62:GFP/agb1-2-1*, and *MYB62:GFP/agb1-2-4* under 100 μM GA_3 treatment at 10d.

Fig. S11 Phenotypes of wild type, *agb-1-2*, *N692967*, *MYB62:GFP/WT-8*, *MYB62:GFP/WT-10*, *MYB62:GFP/agb1-2-1*, and *MYB62:GFP/agb1-2-4* under normal conditions and phosphate-free conditions.

Table S1 Primers used in this study

Table S2 Composition of MS powder

Table S3 Composition of MS powder phosphate-free

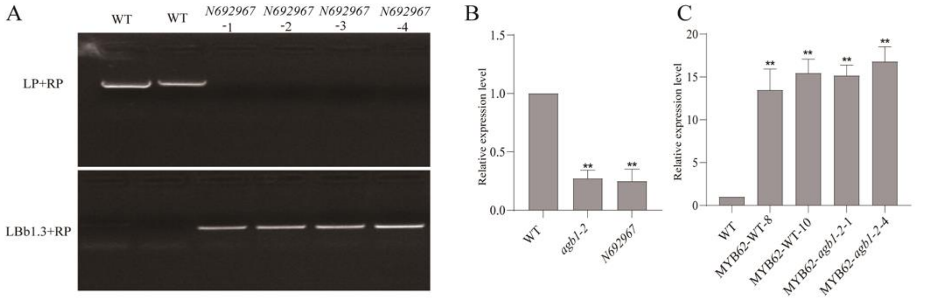

Supplementary Figure S1. Verification of mutants and transgenic plants**.** (A) Genomic PCR verification of AGB1 mutant *N692967*. (B) Analysis of *AGB1* expression in *agb1-2* and *N692967* mutants, grown for four weeks, under normal growth conditions. Data is the average of three independent experiments, and the error bar represents SE (n = 3). Significant differences were analyzed by Duncan's multiple range test ($P < 0.05$). (C) Expression analysis of *MYB62* in transgenic plants *MYB62*:GFP/WT and transgenic plants *MYB62*:GFP/*agb1-2*, grown for four weeks, under normal growth conditions. Data is the average of three independent experiments, and the error bar represents SE (n = 3). Asterisks indicate significant differences between genotypes. Significant differences were analyzed by Duncan's multiple range test ($P < 0.05$).

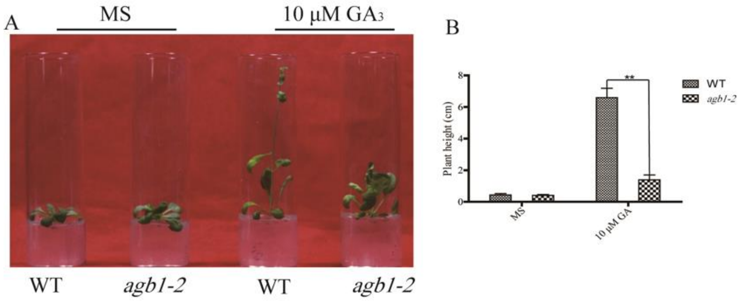

Supplementary Figure S2. Phenotype of mutant *agb1-2* and wild type (*Col-0*) under treatment with 10 µM GA_3. (A) Plant height phenotype of wild type (*Col-0*), *agb1-2* under normal conditions and 10 µM GA_3 treatment. Scale bars, 1 cm. (B) Plant heights of wild type (*Col-0*), *agb1-2* under normal conditions and 10 µM GA_3 treatment. Data is the average of three independent experiments, and the error bar represents SE (n = 10). Asterisks indicate significant differences between genotypes. Significant differences were analyzed by Duncan's multiple range test ($P < 0.05$).

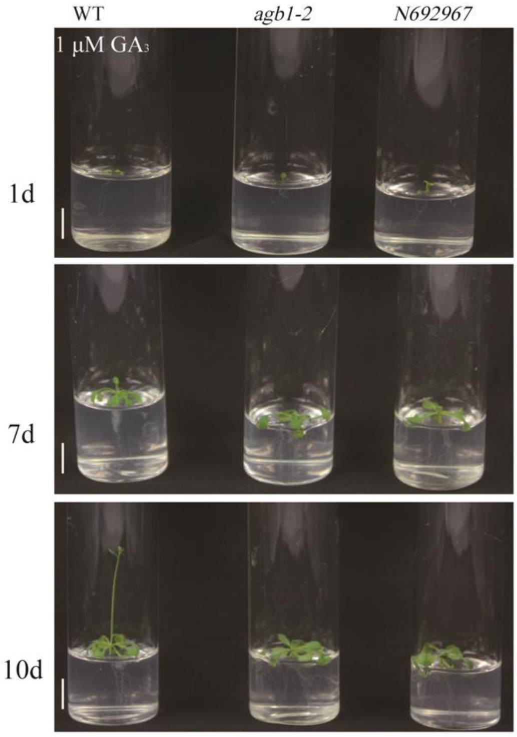

Supplementary Figure S3 Phenotype of wild type, *agb1-2*, and *N692967* under 1 μM GA_3 treatment at 1d, 7d, and 10d. Scale bars, 1 cm.

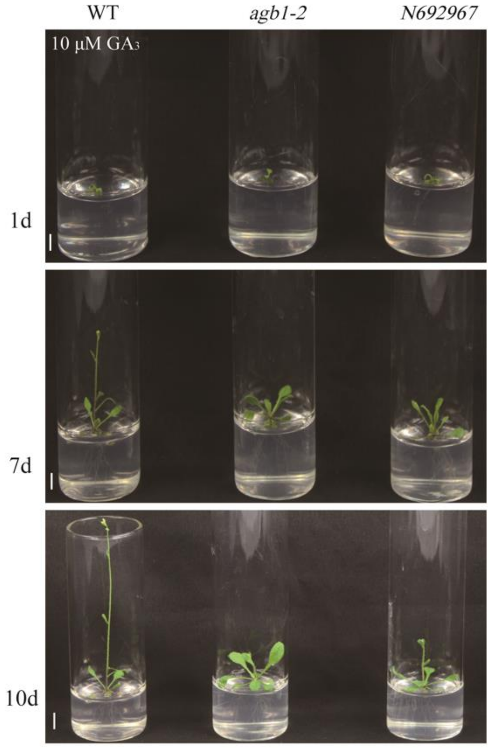

Supplementary Figure S4. Phenotypes of wild type, *agb1-2*, and *N692967* under 10 μM GA_3 treatment at 1 d, 7 d, and 10 d. Scale bars, 1 cm.

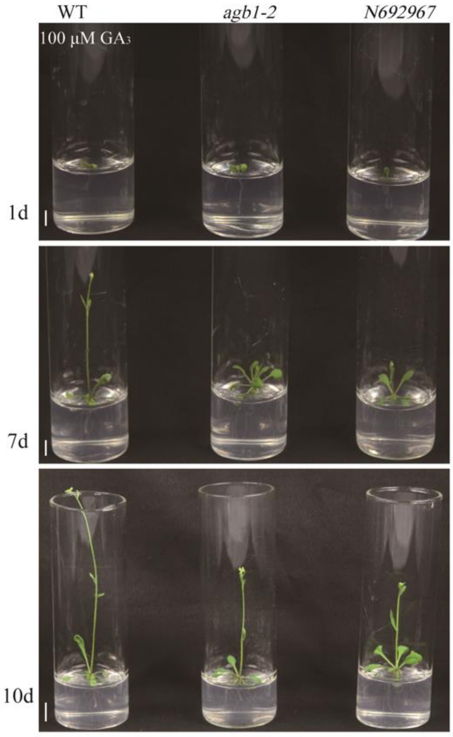

Supplementary Figure S5. Phenotypes of wild type, *agb1-2*, and *N692967* under 100 μM GA_3 treatment at 1 d, 7d, and 10 d. Scale bars, 1 cm.

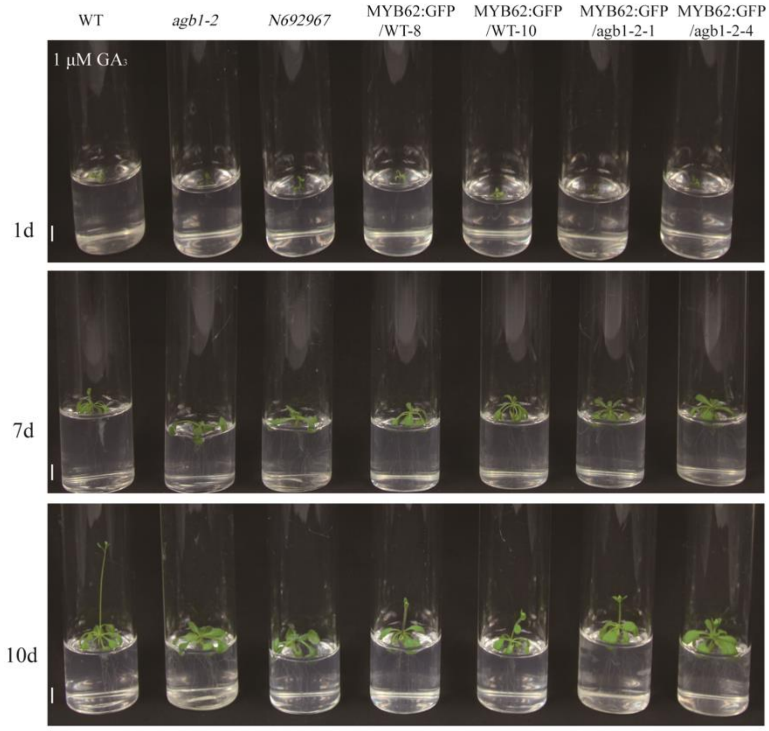

Supplementary Figure S6. Phenotypes of wild type, *agb1-2, N692967, MYB62:GFP/WT-8*, MYB62:GFP/WT-10, *MYB62:GFP/agb1-2-1*, and *MYB62:GFP/agb1-2-4* under 1 μM GA_3 treatment at 1d, 7d, and 10d. Scale bars, 1 cm.

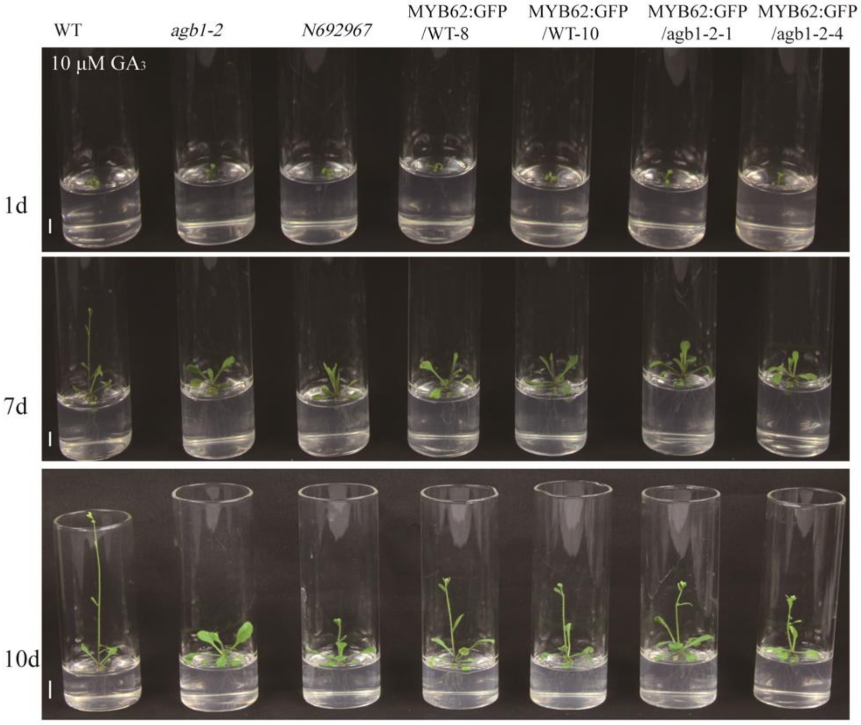

Supplementary Figure S7. Phenotypes of wild type, *agb1-2*, *N692967*, *MYB62:GFP/WT-8*, MYB62:GFP/WT-10, *MYB62:GFP/agb1-2-1*, and *MYB62:GFP/agb1-2-4* under 10 μM GA_3 treatment at 1 d, 7 d, and 10 d. Scale bars, 1 cm.

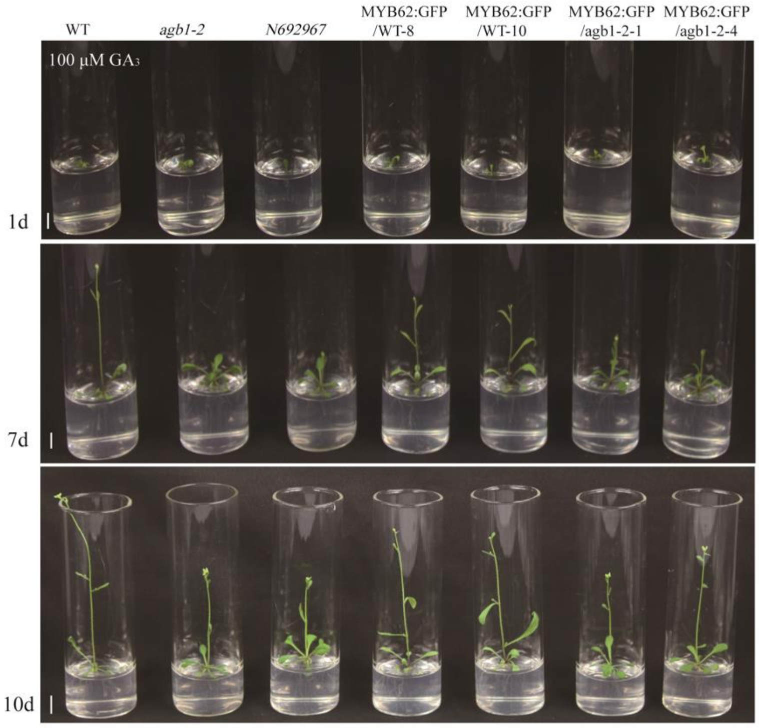

Supplementary Figure S8. Phenotypes of wild type, *agb1-2, N692967, MYB62:GFP/WT-8, MYB62:GFP/WT-10, MYB62:GFP/agb1-2-1,* and *MYB62:GFP/agb1-2-4* under 100 μM GA_3 treatment at 1 d, 7 d, and 10 d. Scale bars, 1 cm.

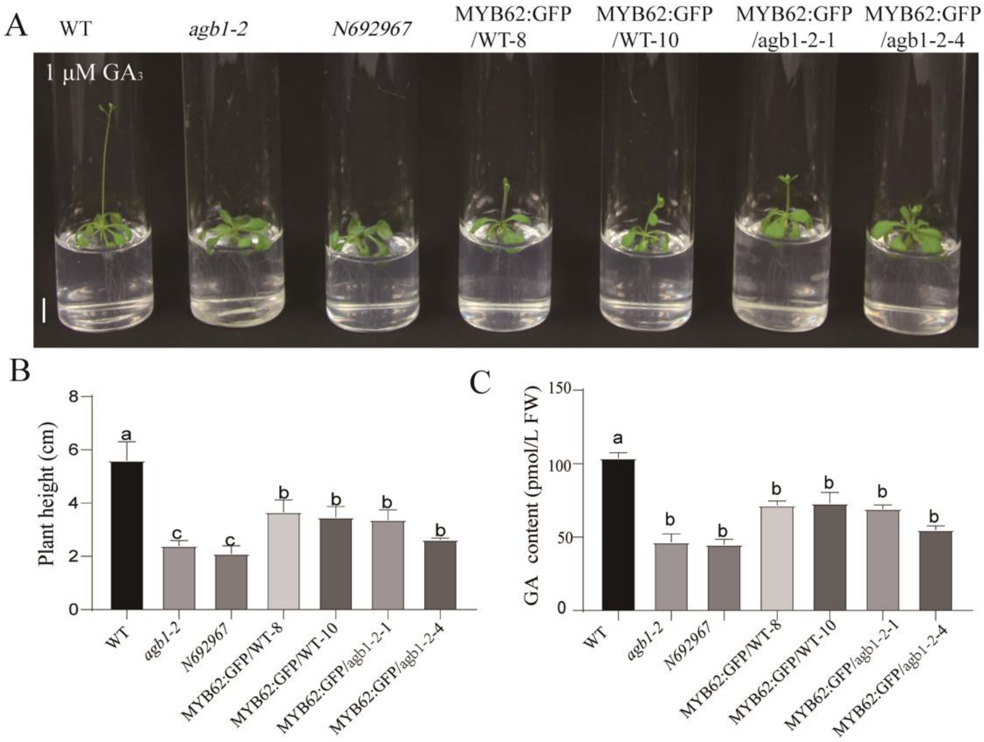

Supplementary Figure S9. Phenotypic identification of WT, *agb1-2*, *N692967*, *MYB62:GFP/WT-8*, *MYB62:GFP/WT-10*, *MYB62:GFP/agb1-2-1*, and *MYB62:GFP/agb1-2-4* under 1 μM GA_3 treatment at 10 d. (A) Plant height phenotypes of wild type, *agb1-2*, *N692967*, *MYB62:GFP/WT-8*, *MYB62:GFP/WT-10*, *MYB62:GFP/agb1-2-1* and *MYB62:GFP/agb1-2-4* following 1 μM GA_3 treatment at 10 d. Scale bars, 1 cm. (B-C) Plant heights and GA contents of wild type, *agb1-2*, *N692967*, *MYB62:GFP/WT-8*, *MYB62:GFP/WT-10*, *MYB62:GFP/agb1-2-1*, and *MYB62:GFP/agb1-2-4* under normal conditions. Data is the average of three independent experiments, and the error bars represent SE (n = 10). Asterisks indicate significant differences between genotypes. Significant differences were analyzed by Duncan's multiple range test ($P < 0.05$).

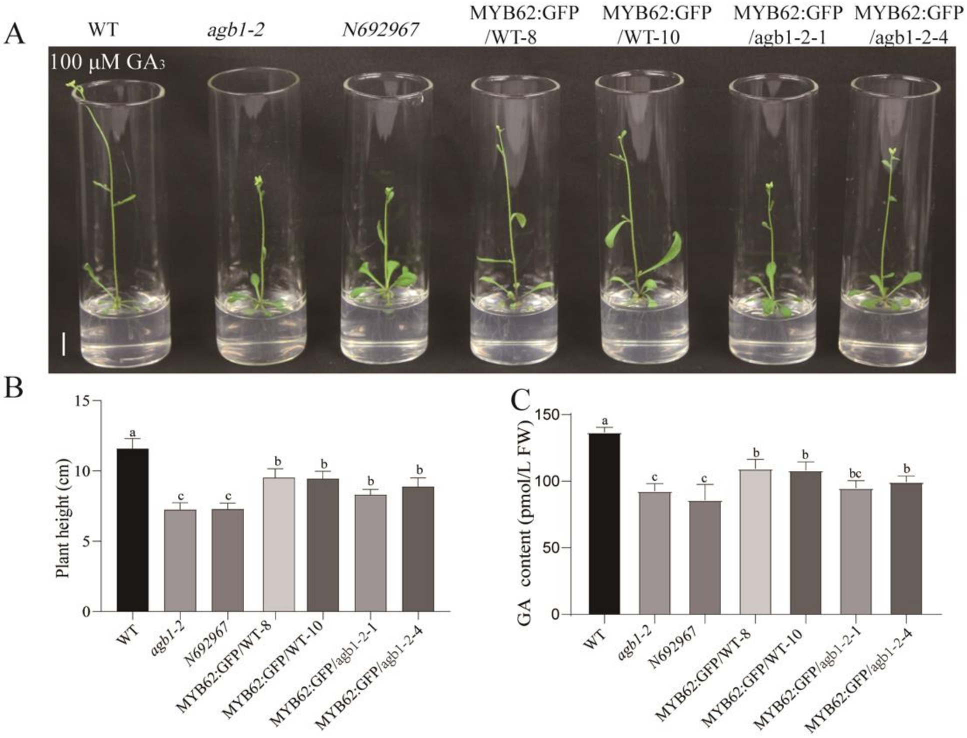

Supplementary Figure S10. Phenotypic identification of WT, *agb-1-2*, *N692967*, *MYB62:GFP/WT-8*, *MYB62:GFP/WT-10*, *MYB62:GFP/agb1-2-1*, and *MYB62:GFP/agb1-2-4* under 100 µM GA_3 treatment at 10 d. (A) Plant height phenotypes of wild type, *agb-1-2*, *N692967*, *MYB62:GFP/WT-8*, *MYB62:GFP/WT-10*, *MYB62:GFP/agb1-2-1*, and *MYB62:GFP/agb1-2-4* following 100 µM GA_3 treatment at 10d. Scale bars, 1 cm. (B-C) Plant heights and GA contents of wild type, *agb-1-2*, *N692967*, *MYB62:GFP/WT-8*, *MYB62:GFP/WT-10*, *MYB62:GFP/agb1-2-1*, and *MYB62:GFP/agb1-2-4* under normal conditions. Data is the average of three independent experiments, and the error bars represent SE (n = 10). Asterisks indicate significant differences between genotypes. Significant differences were analyzed by Duncan's multiple range test ($P < 0.05$).

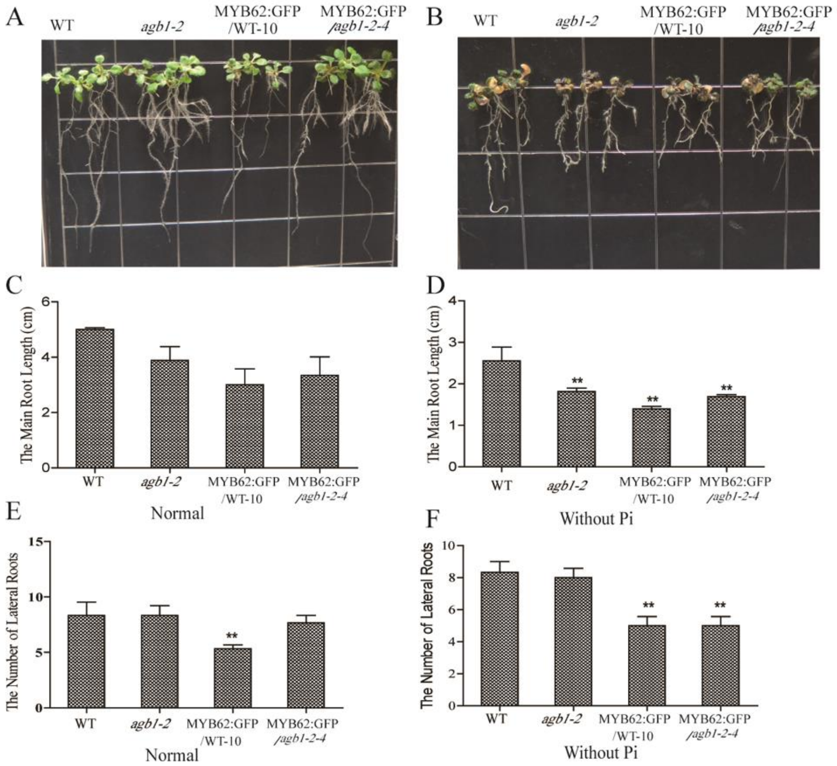

Supplementary Figure S11. Phenotypes of wild type, *agb1-2*, *MYB62:*GFP/WT-*10*, and *MYB62:*GFP*/agb1-2-4* under normal conditions and phosphate-free conditions. (A) Phenotype identification of wild type, *agb1-2* mutant, *MYB62*-GFP/WT-*10*, and *MYB62*-GFP*/agb1-2-4* under normal conditions. Bar = 1cm. (B) Phenotype identification of wild type, *agb1-2* mutant, *MYB62*-GFP/*WT-10*, and *MYB62*-GFP/*agb1-2-4* under phosphate-free conditions. Bar = 1cm. (C) The main root lengths of wild type, *agb1-2* mutant, *MYB62*-GFP/*WT-10*, and *MYB62*-GFP/*agb1-2-4* under normal conditions. Error bars represent the means ± SE (n = 3). Asterisks indicate significant differences between genotypes. Significant differences were analyzed by Duncan's multiple range test (P < 0.05). (D) The numbers of lateral roots of the wild type, *agb1-2* mutant, *MYB62-GFP/WT-10*, and *MYB62-GFP/agb1-2-4* under normal conditions. Error bars represent the means ± SE (n = 3). Significant differences were analyzed by Duncan's multiple range test (P < 0.05). (E) The main root lengths of wild type, *agb1-2* mutant, *MYB62-GFP/WT-10*, and *MYB62-GFP/agb1-2-4* under phosphorus-free conditions. Error bars represent the means ± SE (n = 3). Asterisks indicate significant differences between genotypes. Significant differences were analyzed by Duncan's multiple range test (P < 0.05). (F) The numbers of lateral roots of wild type, *agb1-2* mutant, *MYB62-GFP/WT-10*, and *MYB62-GFP/agb1-2-4* under phosphorus-free conditions. Error bars represent the means ± SE (n = 3). Asterisks indicate significant differences between genotypes. Significant differences were analyzed by Duncan's multiple range test (P < 0.05).

Table S1 Primers used in this study

**Name**	**Primer sequence (5’-3’)**
**Primers used for qRT-PCR**	
qRT-AtAGB1-F	ACGTTTGGGATACCCTCTTGG
qRT-AtAGB1-R	GTGTCCTCCAAACGCCCATA
qRT-AMYB62-F	TGGCTCGATGTGTTTCCATGA
qRT-AtMYB62-R	ACGCTGAATAACCATCCGCC
qRT-AtGA2ox7-F	AGTGGTGAGGAGGTCAAACG
qRT-AtGA2ox7-R	GAGAAGTGGCGCTAGGGTTT
qRT-AtGA2ox4-F	TGTCCGACCAGTTAACCAGC
qRT-AtGA2ox4-R	GGCAGGGTCGTTAGTGTGAA
qRT-AtGAMT1-F	ATGGAGTCGTCACGGAGCC
qRT-AtGAMT1-R	TGGAGTTGATGGCTGTCGTC
**Primers used to generate DNA constructs for the yeast two-hybrid assay**	
AGB1-BD-F	AGGAGGACCTGCATATGATGTCTGTCTCCG
AGB1-BD-R	GCCTCCATGGCCATATG AATCACTCTCCTG
MYB62-AD-F	CATCGATACGGGATCCATGGAAAATTCGA
MYB62-AD-R	CGAGCTCGATGGATCCATCTCCCTAAACTG
**Primers used to generate DNA constructs for the subcellular localization assay**	
AGB1-16318-F	TATCTCTAGAGGATCCATGGGCTTACTCTGC
AGB1-16318-R	TGCTCACCATGGATCCAATCACTCTCCTGT
MYB62-16318-F	TATCTCTAGAGGATCCATGGAAAATTCGAT
MYB62-16318-R	TGCTCACCATGGATCCCTCCCTAAACTGCC
**Primers used to generate DNA constructs for protein expression**	
AGB1-GST-FULL-F	CGGGATCCATGTCTGTCTCCGAGCTCAAAG
AGB1-GST-FULL-F	GCGTCGACAATCACTCTCCTGTGTCCTC
MYB62-MBP-F	CGGGATCCATGTCTACCGATGTGGCGAGGT
MYB62-MBP-R	GCGTCGACAATCACTCTCCTGTGTCCTC
**Primers used to generate DNA constructs for LUC assay**	
AGB1-nLUC-F	TCGGTACCCGGGATCCATGTCTGTCTCCGAG
AGB1-nLUC-R	CCATTTGTTGGATCCAATCACTCTCCTGTG
MYB62-cLUC-F	CGGGGCGGTACCATGGAAAATTCGA
MYB62-cLUC-R	GTTGCTGCAGGTCGACCTCCCTAAACTG
**Primers used to generate DNA constructs for Co-IP assays**	
AGB1-Flag-F	GGGGCCCGGGGTCGACATGTCTGTCTCCGAGCTCAAAG
AGB1-Flag-R	TACCGGATCCACTAGTAATCACTCTCCTGTGTCCTC
MYB62-GFP-F	GATTACGAATTCATGGAAAATTCGATG

MYB62-GFP-R	GTGCTCGAATTCCTCCCTAAACTGCCAAATG
**Primers used for genotyping**	
AGB1-F (LP)	TCATTAGATTGGACACCGGAG
AGB1-R (RP)	TGTGAATCCTGCTGTAATCCC
MYB62-F (P1)	TGAGATCAATCGGCTAAAAGC
MYB62-R (P2)	CAAAGATTGCGATTCATCGAT
LBb1.3	ATTTTGCCGATTTCGGAAC
**Primers used to produce transgenic plants**	
OE-MYB62-F	GGGGCCCGGGGTCGACATGGAAAATTCGATGAAGAA GAAGA
OE-MYB62-R	TACCGGATCCACTAGTCC CTCCCTAAACTGCCAAATGTCAT
**Primers used to identify the homozygote *N692967* mutants**	
LP	TCATTAGATTGGACACCGGAG
RP	TGTGAATCCTGCTGTAATCCC
LB	ATTTTGCCGATTTCGGAAC
**Primers used for EMSA analysis**	
ProGA2ox7-F	BIO-AAGCCCTTGGTTGGGGATGG
ProGA2ox7-R	BIO-CCATCCCCAA CCAAGGGCTT
**Primers used to generate DNA constructs for LUC assays**	
GA2ox7-F	GCAGCCCGGGGGATCCTCCATTACTAGAGAA
GA2ox7-R	TAGAACTAGTGGATCCTAAACTGAAT TAAGT

Table S2 Composition of MS medium

**Component**		
**Macro elements**	**mg/L**	**mM**
$CaCl_2$	332.02	2.99
KH_2PO_4	170.00	1.25
KNO_3	1900.00	18.79
$MgSO_4$	180.54	1.50
NH_4NO_3	1650.00	20.61
**Micro elements**	**mg/L**	**µM**
$CoCl_2 \cdot 6H_2O$	0.025	0.11
$CuSO_4 \cdot 5H_2O$	0.025	0.10
FeNaEDTA	36.70	100.00
H_3BO_4	6.2	100.27
KI	0.83	5.00
$MnSO_4 \cdot H_2O$	16.90	100.00
$Na_2MoO_4 \cdot 2H_2O$	0.25	1.03

$ZnSO_4$•$7H_2O$	8.60	29.91
**Vitamins**	**mg/L**	**µM**
Glycine (free base)	2.00	26.64
myo-Inositol	100.0	554.94
Nictotinic acid (free acid)	0.50	4.06
Pyridoxine • HCl	0.50	2.43
Thiamine • HCl	0.10	0.30

Table S3 Composition of MS phosphate-free medium

**Component**		
**Macro elements**	**mg/L**	**mM**
$CaCl_2$	332.02	2.99
KNO_3	1900.00	18.79
$MgSO_4$	180.54	1.50
NH_4NO_3	1650.00	20.61
**Micro elements**	**mg/L**	**µM**
$CoCl_2$ • $6H_2O$	0.025	0.11
$CuSO_4$ • $5H_2O$	0.025	0.10
FeNaEDTA	36.70	100.00
H_3BO_4	6.2	100.27
KI	0.83	5.00
$MnSO_4$•H_2O	16.90	100.00
Na_2MoO_4•$2H_2O$	0.25	1.03
$ZnSO_4$•$7H_2O$	8.60	29.91
**Vitamins**	**mg/L**	**µM**
Glycine (free base)	2.00	26.64
myo-Inositol	100.0	554.94
Nictotinic acid (free acid)	0.50	4.06
Pyridoxine • HCl	0.50	2.43
Thiamine • HCl	0.10	0.30
